# Supplementary figures and images for: Pigment cell progenitor heterogeneity and reiteration of developmental signaling underlie melanocyte regeneration in zebrafish
Source: eLife. 2023 Apr 6;12:e78942. doi: 10.7554/eLife.78942 (PMC10139689; doi:10.7554/eLife.78942)

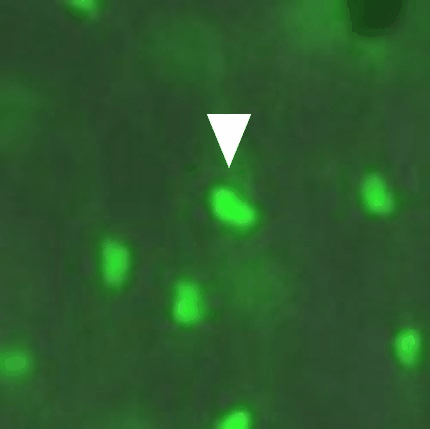

Supplement: Supplementary file 11 [file elife-78942-video1.gif]

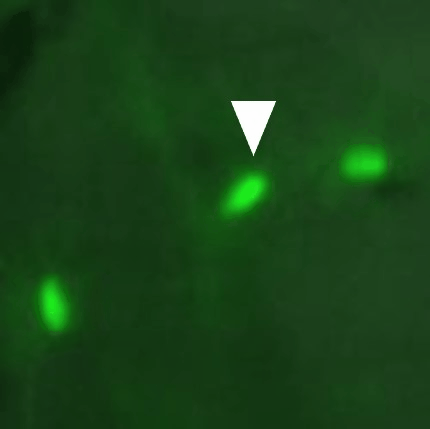

Supplement: Supplementary file 12 [file elife-78942-video2.gif]
